# Mepolizumab treatment alters the functional phenotype of eosinophils in severe eosinophilic asthma

**DOI:** 10.3389/fimmu.2025.1654111

**Published:** 2025-11-27

**Authors:** Pablo Miguéns-Suárez, Laura Martelo-Vidal, Sara Vázquez-Mera, Marina Blanco-Aparicio, Uxío Calvo-Álvarez, Coral González-Fernández, Mar Mosteiro-Añón, Dolores Corbacho-Abelaira, Tamara Hermida-Valverde, Christian Calvo-Henríquez, Juan J. Nieto-Fontarigo, Francisco J. Salgado, Francisco J. González-Barcala

**Affiliations:** 1 Translational Research in Airway Diseases Group (TRIAD)-Health Research Institute of Santiago de Compostela (IDIS), Santiago de Compostela, Spain; 2 Department of Biochemistry and Molecular Biology, Faculty of Biology-Biological Research Centre (CIBUS), Universidade de Santiago de Compostela, Santiago de Compostela, Spain; 3 Department of Respiratory Medicine, University Hospital of A Coruña, A Coruña, Spain; 4 Department of Respiratory Medicine, University Hospital of Santiago de Compostela, Santiago de Compostela, Spain; 5 Department of Respiratory Medicine, University Hospital of Ourense, Ourense, Spain; 6 Department of Respiratory Medicine, University Hospital Álvaro Cunqueiro, Vigo, Spain; 7 Department of Respiratory Medicine, Ribera Hospital POVISA of Vigo, Vigo, Spain; 8 Department of Respiratory Medicine, Central University Hospital of Asturias (HUCA), Oviedo, Spain; 9 Department of Otorhinolaryngology, University Hospital of Santiago de Compostela, Santiago de Compostela, Spain; 10 Department of Medicine, University of Santiago de Compostela, Santiago de Compostela, Spain

**Keywords:** anti-IL-5 mAb, biologic, eosinophil activation, eosinophil subsets, IL-5, severe asthma

## Abstract

**Background:**

Blood eosinophil count is usually employed as a predictive and response biomarker for mepolizumab treatment. However, its decrease is not always associated with an improvement in asthma symptoms. The aim of this work is to study the effect of mepolizumab in the activation status and functional phenotype of circulating eosinophils.

**Methods:**

Samples from healthy controls (N = 15) and patients with severe eosinophilic asthma (N = 15) before and after 4, 16 and 32 weeks of mepolizumab treatment (anti-IL5 mAb; 100 mg s.c./4 weeks) were screened. Demographic, clinical, hematological and biochemical characteristics were collected. Activation and functional phenotype of peripheral blood eosinophils was analyzed by flow cytometry. sCD62L in serum was measured by ELISA. Target mRNAs were quantified by Nanostring.

**Results:**

Eosinophils from severe eosinophilic asthma patients showed a higher activation profile (CD11b, CD44 and IL-5Rα expression) compared to healthy subjects. Mepolizumab treatment reduced the number of basophils and eosinophils in peripheral blood. We also found a clear downmodulation on the surface expression (% and MFI) of CD44 and IL-3Rα on eosinophils at week 4, which was maintained through all treatment period (4–32 weeks). The functional phenotype of the remaining eosinophils was also modified with the treatment, showing a reduction of inflammatory eosinophils (iEOS; CD62L^lo^) percentage without affecting the balance of regulatory subpopulations (CD16^dim/hi^ eosinophils). This was accompanied by a decrease in serum sCD62L levels. mRNA and protein levels showed similar trends for some targets (e.g., IL-5Rα) but not for others (e.g., CD62L).

**Conclusions:**

Mepolizumab treatment modifies the functional phenotype of eosinophils resulting in a lower percentage of iEOS and reduced activation status. These changes occur at an early time point (4 weeks) and are maintained throughout all treatment period.

## 1 Introduction

Asthma is a chronic inflammatory disease with several phenotypes and severity degrees (1). Five to ten percent of the patients develop severe asthma, a disease that remains uncontrolled despite maximum ICS/LABA therapy. Severe asthma patients had a declined lung function and frequent disease exacerbations leading to a high morbidity, mortality, and socioeconomic burden (1). Interestingly, around 80% of these patients refer increased numbers of eosinophils in blood and airways (i.e., severe eosinophilic asthma/SEA) (2).

SEA is part of the T2-high molecular phenotype and usually represents an independent group or endotype in clustering studies from different asthma cohorts (e.g., SARP (3), U-BIOPRED (4)). This endotype is commonly associated with patients with adult-onset, frequent exacerbations, and poor prognosis (5). In addition, patients with SEA have a higher prevalence of chronic rhinosinusitis, nasal polyposis, and persistent airflow limitation with air trapping (5). Previous studies demonstrated that type-2 cytokines (i.e., IL-4, IL-5, and IL-13) play critical roles in the pathogenesis of SEA (6). Particularly, IL-5 participates in many levels of eosinophil biology, including their chemotaxis, activation, proliferation, differentiation, maturation, degranulation, and enhanced survival (7). Thus, targeting IL-5 pathway has been described as a recommended approach to treat SEA patients (7).

Mepolizumab (Nucala^®^, SB-240563, GlaxoSmithKline) is an IL-5 antagonist monoclonal antibody approved for SEA patients (1). Several studies (8–12), two of them from our group (13, 14), reported a reduction in the number of exacerbations, the need for oral corticosteroids, as well as an improvement in asthma control and quality of life after mepolizumab treatment in patients with SEA. At cellular level, the most studied and characterized effect of mepolizumab is the prolonged sharp decrease of eosinophil number in peripheral blood and induced sputum (8–14).

Although blood eosinophil count is usually employed as a predictive and response biomarker for mepolizumab treatment, its decrease is not always associated with an improvement in asthma symptoms (15). Therefore, it is important to study not only the number, but the functional phenotype of eosinophils after therapy. Aligned with this, several proteins have been described as biomarkers of eosinophil activation, including CD44, integrin αM (CD11b), or several cytokine receptors (i.e., IL-3Rα/CD123 and IL-5Rα/CD125)] (16, 17). In addition, different subtypes of eosinophils have been defined attending to their surface markers: regulatory/suppressive eosinophils (SSC^hi^CCR3^+^CD16^hi^) (18), and the resident (CD62L^hi^) and inflammatory (CD62L^lo^) eosinophils (19, 20).

The aim of our work is to analyze the effect of mepolizumab on the proportions of circulating eosinophil subpopulations and the molecular/functional phenotype of the remaining eosinophils. This could lead to the discovery of new response and predictive biomarkers, which may help to monitor patient evolution and to optimize the profile of patients starting treatment.

## 2 Materials and methods

### 2.1 Study population

**Figure 1:**
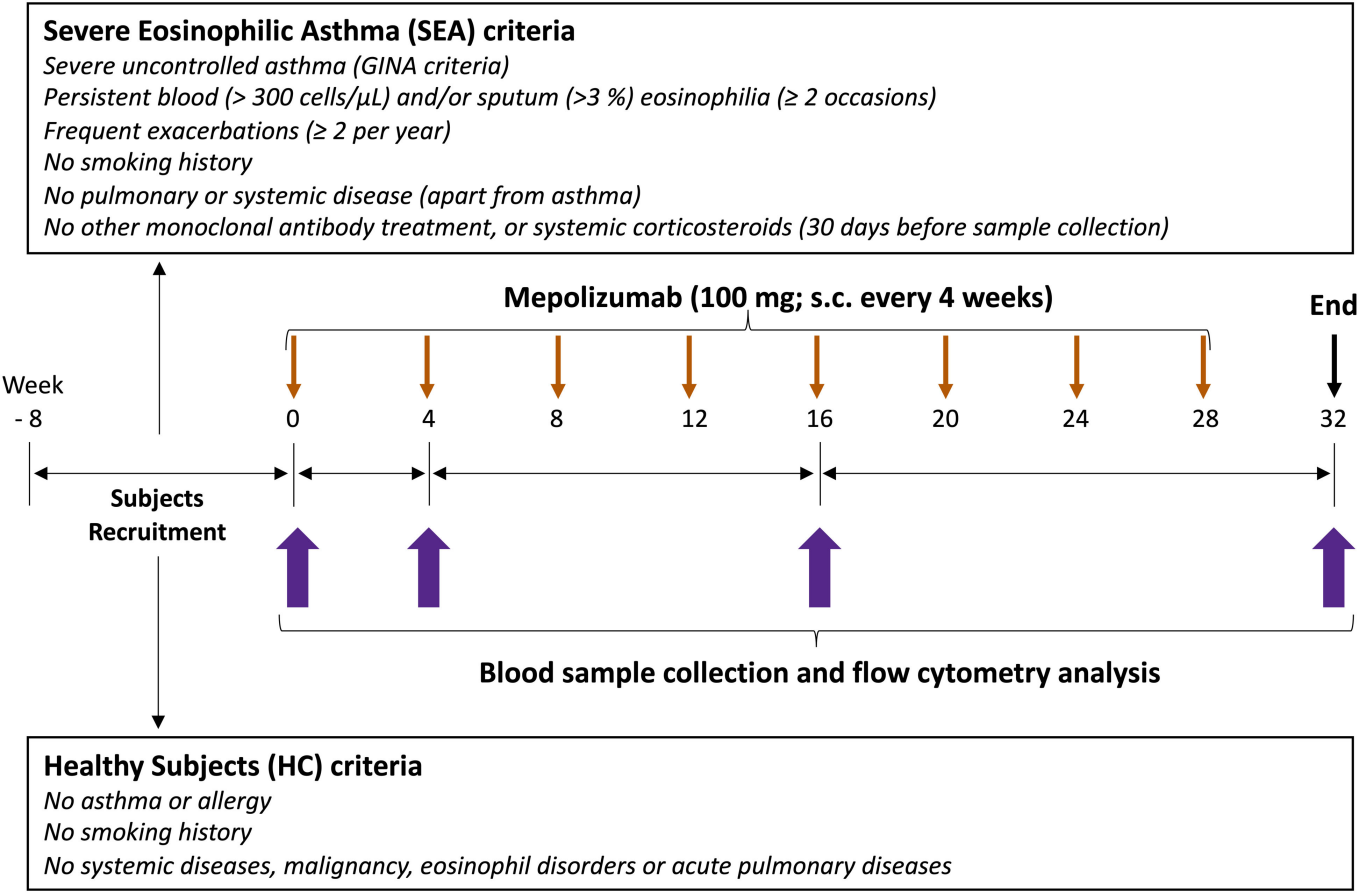
Study design. The first part of the study was an observational case-control study. A reference group of healthy subjects (HC, N = 15), and adult patients with severe eosinophilic asthma (SEA) selected for treatment with mepolizumab (N = 15) were recruited. The second part of the study was a post-authorization, observational cohort study. SEA patients underwent a subcutaneous injection of mepolizumab (100 mg) every 4 weeks during the whole study period (32 weeks); totally 8 doses. Clinical and demographic data, together with a venous peripheral blood sample were collected at inclusion (week 0, baseline) for both SEA and HC groups, and 4, 16 and 32 weeks (T0, T4, T16 and T32) after mepolizumab treatment only in SEA group.

Study design is depicted in **Figure 1**. A set of 15 healthy controls (HC) and 15 SEA patients with a similar age and sex proportion were included in EMESEA.ppt project (ClinicalTrials.gov ID: NCT04641741). EMESEA.ppt is a multicentric study and SEA patients were recruited from 6 different hospitals from NW Spain. Inclusion criteria for SEA patients were as follows: diagnosis of severe uncontrolled asthma according to ERS/ATS criteria (see Supplementary Methods); persistent eosinophilia in blood (≥ 300 cells/μL) on ≥ 2 occasions separated by at least 4 weeks; and frequent exacerbations (≥ 2 per year, defined as ≥ 3 days of lack of asthma control requiring systemic corticosteroids and/or ED visit and/or hospitalization).

HC participants were recruited from the anesthesia service of the University Hospital Complex of Santiago de Compostela. HC were excluded based on the presence of respiratory and immune conditions [history of asthma or any pulmonary disease; disorders associated with elevated eosinophils (e.g., ABPA, Churg-Strauss syndrome, hypereosinophilic syndrome)]. In addition, the following exclusion criteria were applied to both HC and SEA patients: medications affecting the immune system [recent treatment with systemic corticosteroids, biologics (e.g., omalizumab or other monoclonal antibodies), or immunosuppressive drugs]; smoking (current smokers or former smokers with ≥ 10 pack-years); systemic disorders (unstable cardiovascular, hepatic, renal, neurological, musculoskeletal, infectious, endocrine, metabolic, hematological, psychiatric, or other major conditions); malignancy (current cancer or history of cancer in remission); recent infections (acute upper or lower respiratory infection within 30 days prior to sampling); pregnancy or breastfeeding; and obesity [class 2 or higher (BMI ≥ 35 kg/m^2^)].

SEA patients underwent a subcutaneous injection of mepolizumab (100 mg) every 4 weeks during the whole study period (32 weeks); a total of 8 doses. Demographic data was collected at inclusion. Clinical data was also collected in SEA patients before and after treatment, including ACT control questionnaire, lung function (FEV1%, FEV1/FVC), FeNO, IgE levels, and hematological counts. Blood samples were collected at inclusion (week 0, baseline) for both SEA and HC groups, and 4, 16 and 32 weeks (T0, T4, T16 and T32) after mepolizumab treatment only in SEA group. Three T32 samples are missed due to patients failed to attend their scheduled consultations.

All participants signed a written informed consent. The study was approved by the Committee on Ethics of Research with medicines of Galicia (CEIm-G; Ref. 2020/406) and Helsinki Declaration was followed.

### 2.2 Flow cytometry analysis

Blood samples were collected in BD Vacutainer K2 EDTA tubes via venepuncture. A hundred µL of blood were stained with different antibodies (**Supplementary Table S1**) for 20 minutes. Erythrocytes were lysed using BD FACS Lysing Solution. Cells were washed twice with PBS containing 1% BSA, 1mM EDTA and 0.05% sodium azide.

Eosinophils were gated as SSC^hi^CCR3^+^ cells, and the % of positive cells and median fluorescence intensity (MFI) values for different surface proteins were measured using a BD FACSort Flow Cytometer. A complete gating strategy is depicted in **Supplementary Figure S1**. Flow cytometry data was analyzed using Flowjo 10.4.0.

### 2.3 Human serum CD62L ELISA

sCD62L was measured in serum samples using the human CD62L ELISA kit (R&D Systems, Minneapolis, MI, cat. no DY728) in accordance with the manufacturer’s instructions.

### 2.4 Eosinophil isolation and RNA purification

Eosinophils were purified from 30 mL of peripheral blood [N = 9; only before (T0) and after 16 weeks of mepolizumab (T16)]. Dextran sedimentation of samples was carried out to remove erythrocytes. Eosinophils were isolated with the EasySep™ Human Eosinophil Isolation kit according to manufacturer instructions. An additional final wash with the buffer without FBS was made before cell lysis. Isolated eosinophils were lysed and RNA was extracted using a column-based purification workflow [Nucleospin™ RNA/Protein kit] according to manufacturer instructions. RNA was further cleaned and concentrated with RNA Clean & Concentrator™ kit and samples were stored at -80 °C before initiating gene expression analysis. Eosinophil purity was determined based on morphology as > 95% by Kwik-Diff staining.

### 2.5 Nanostring nCounter gene expression analysis

RNA sample degradation was assessed using a 4200 TapeStation System and concentration was determined using Qubit 4 Fluoremeter. 100 ng of RNA were used for Nanostring analysis. Gene expression profiling was performed using the Nanostring nCounter Sprint equipment with selected genes from a panel of 770 genes (nCounter Human Myeloid Innate Immunity V2 Panel). Hybridization was carried out according to the manufacturer instructions. Data acquisition was performed on the nCounter Digital Analyzer. Raw counts were normalized using top 18 stable housekeeping genes.

### 2.6 Data and statistical analysis

Analysis was performed using GraphPad Prism 10.2.2 (GraphPad Software, La Jolla, CA, USA). Mann-Whitney U test was used to compare data between HC and SEA groups. A mixed-effects analysis with the Geisser-Greenhouse correction followed by Holm-Šídák’s multiple comparisons test was used to address the effect of mepolizumab treatment at different time-points. Spearman´s rank correlation was used to assess correlation between variables. Normalized gene expression was log_2_ transformed. A paired differential expression analysis comparing T0 and T16 was performed using a moderated t-test inside limma R package. p-values were adjusted for multiple testing using the Benjamini-Hochberg correction. Statistical significance was determined as a p-value < 0.05.

## 3 Results

### 3.1 Clinical characteristics of study population

Baseline characteristics of participants are shown in **Table 1**. SEA patients and HC had a similar age and sex proportion (**Table 1**). Pulmonary function parameters FEV1 (%) and FEV1/FVC ratio for SEA patients were 86.7 ± 25.4 and 69.3 ± 11.8, respectively. Asthma control at inclusion was poor, with ACT values of 13.4 ± 4.6 (**Table 1**), despite all of them were treated with ICS/LABA. At baseline, SEA patients displayed higher eosinophil, basophil, and monocyte counts compared to HC individuals (**Table 1**). No other changes were found in the remaining blood leukocytes populations.

**Table 1:** Demographic and clinical characteristics of trial participants at baseline.

Variables	HC	SEA	p-value
N	15	15	-
Demographic data			
Age, years	55.5 (12.6)	57.1 (12.1)	ns
Gender (M/F)	3/12	3/12	ns
Pulmonary function			
FEV1%	-	87.8 (24.9)	-
FVC %		99.1 (19.4)	
FEV1/FVC ratio	-	69.6 (11.5)	-
ACT	-	13.4 (4.6)	-
FeNO	-	60.6 (60.3)	-
Hematological variables			
Eosinophils (cells/μL)	190.0 (94.6)	540.7 (248.8)	<0.0001
(%)	3.2 (1.3)	7.6 (2.9)	<0.0001
Neutrophils (cells/μL)	3250.7 (881.1)	3812.7 (982.8)	ns
(%)	54.0 (8.0)	51.8 (7.7)	ns
Lymphocytes (cells/μL)	2090.0 (717.0)	2410.0 (667.9)	ns
%	34.5 (6.8)	33.1 (7.1)	ns
Monocytes (cells/μL)	335.7 (104.2)	522.1 (177.9)	0.0026
%	5.6 (1.4)	7.2 (2.2)	ns
Basophils (cells/μL)	37.1 (19.0)	60.7 (21.6)	0.0052
%	0.60 (0.25)	0.8 (0.3)	0.0171

N, number of cases; M, male; F, female; Abs, absolute count of blood leukocytes (cells/μL).

Mean (SD) of the variables is represented.

Mann-Whitney U test was used to compare significant differences for continuous variables.

Chi-squared test was used to compare gender proportion.

p-values of significant differences are shown in the right column.

### 3.2 Increased eosinophil activation in patients with SEA

**Figure 2:**
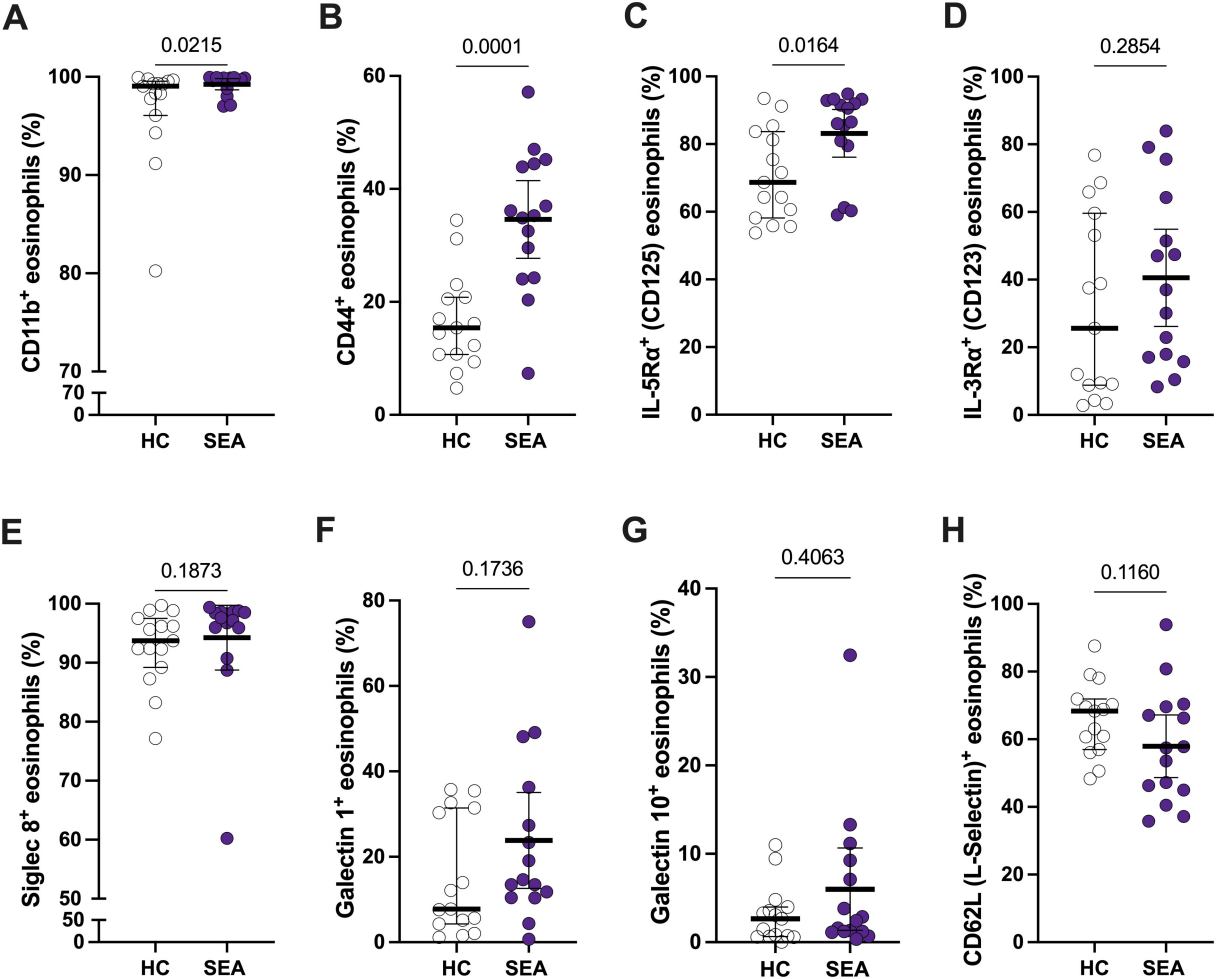
Peripheral blood eosinophils from SEA patients show a different activation profile compared to HC. The percentage of CD11b^+^**(A)**, CD44^+^**(B)**, IL-5Rα^+^**(C)**, IL-3Rα^+^**(D)**, Siglec-8^+^**(E)**, galectin-1^+^**(F)**, galectin-10^+^**(G)**, and CD62L (L-selectin)^+^**(H)** eosinophils is depicted. Mann-Whitney U test; p-value is shown for each comparison.

Apart from an increase in the number of eosinophils in SEA (**Table 1**), changes in the activation pattern and function of those cells might be associated with asthma. Thus, we first evaluated the eosinophil expression patterns of different activation markers, including CD11b (αM integrin), CD44, IL-5Rα (CD125), and IL-3Rα (CD123), between HC and SEA subjects. Additionally, surface proteins potentially involved in eosinophil regulatory function (e.g., galectins), Siglec-8 (a late differentiation marker of eosinophils), and CD62L (L-selectin) were also compared (**Figure 2**).

The percentage of CD11b^+^ and CD44^+^ eosinophils was higher in SEA patients compared to HC (**Figures 2A, B**). Regarding the cytokine receptors, only IL-5Rα^+^ % was significantly increased in SEA vs. HC (**Figures 2C, D**). Furthermore, this % of IL-5Rα^+^ eosinophils is higher than 80% in most of SEA patients (**Figure 2C**), making them a suitable target for mepolizumab treatment. A similar trend in activation markers was found for MFI values, but only significant for CD44 (**Supplementary Figures S2A–D**). The expression of galectin-1 and galectin-10, Siglec-8, or CD62L was not significantly changed between HC and SEA patients (**Figures 2E–H**; **Supplementary Figures S2E-H**).

### 3.3 Mepolizumab treatment decreased the number and activation status of eosinophils in SEA

Taking into account that eosinophil activation was increased in SEA, including a higher proportion of expressing IL-5Rα eosinophils, we next aimed to determine the effect of blocking IL-5 on the expression pattern of the eosinophilic activation markers exposed above. SEA patients included in the study received mepolizumab (100 mg, s.c., every 4 weeks) for 32 weeks (**Figure 1**). This resulted in a clear improvement of asthma control, with ACT increasing from 13.4 to 23.8 at week 32 (p < 0.0001) (**Supplementary Table S2**). However, no changes were found in pulmonary function test (i.e., FEV1% and FEV1/FVC%), or FeNO (**Supplementary Table S2**) with the treatment.

**Figure 3:**
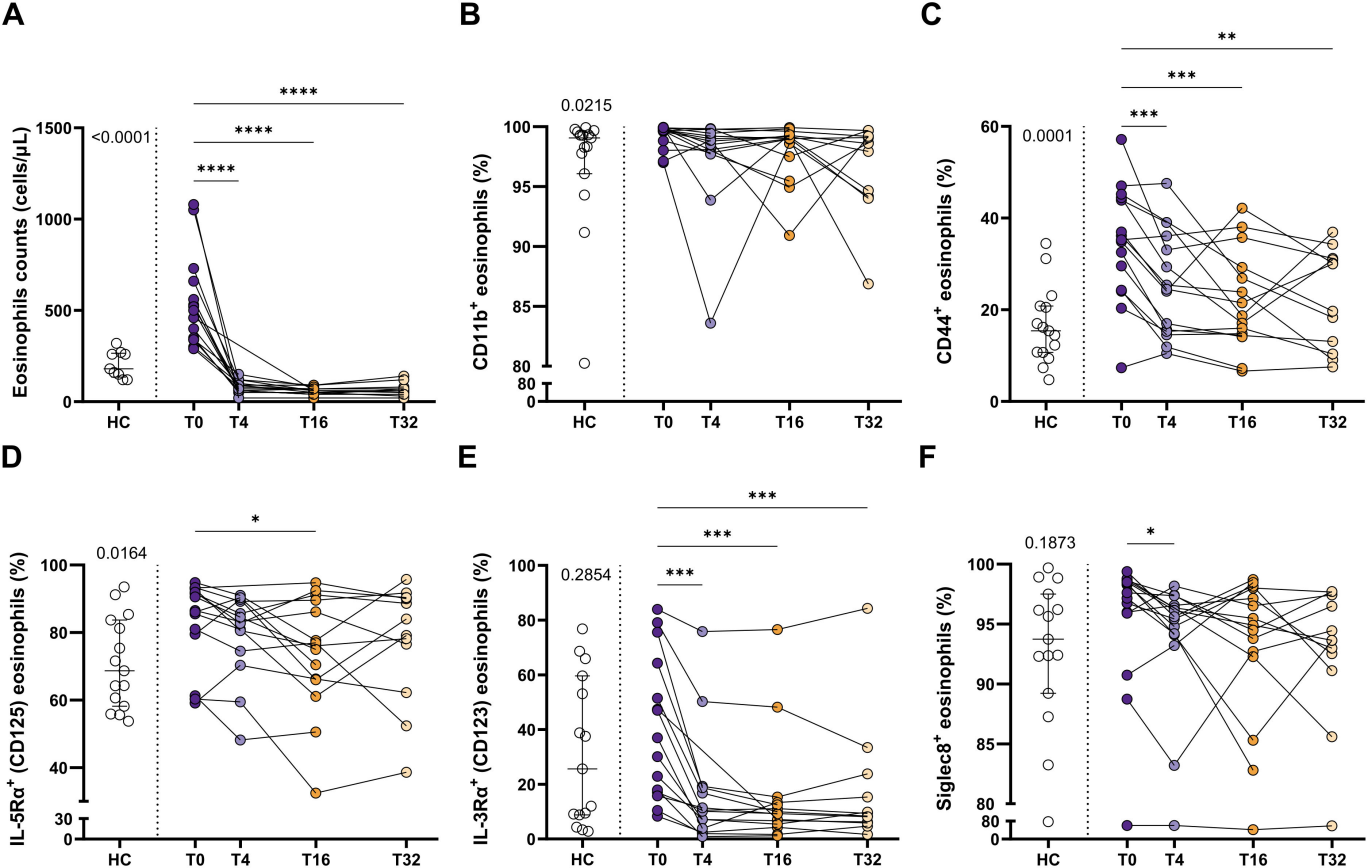
Peripheral blood eosinophils have a lower activation status after mepolizumab treatment. **(A)** Number of eosinophils in peripheral blood. **(B–F)** Percentage of CD11b^+^**(B)**, CD44^+^**(C)**, IL-5Rα^+^**(D)**, IL-3Rα^+^**(E)**, and Siglec-8^+^**(F)** eosinophils in healthy (HC) and SEA before (T0) and after 4 (T4), 16 (T16), and 32 (T32) weeks of mepolizumab treatment. Mann-Whitney U test to compare HC vs SEA; p-value is shown for each comparison. A mixed-effects analysis with the Geisser-Greenhouse correction followed by Holm-Šídák’s multiple comparisons test was used to address the effect of mepolizumab treatment at different time-points; *p < 0.05; **p < 0.01; ***p < 0.001.

Regarding hematological cell counts, mepolizumab reduced both the number and percentage of eosinophils and basophils (**Supplementary Table S2**). Remarkably, the number of eosinophils in treated patients is significantly lower than those from healthy individuals (**Figure 3A**). Moreover, this decrease was maximum at week 4 (T4) and maintained during the whole period evaluated (T4-T32; **Figure 3A**). Eosinophil activation was also affected by the treatment (**Figures 3B–E**; **Supplementary Figure S3**). Thus, CD44^+^ and IL-3Rα^+^ eosinophils were markedly reduced after mepolizumab at T4, and also maintained at week 16 and 32 (**Figures 3C, E**). MFI values were affected in the same manner, including also a downmodulation of CD11b at T32 (**Supplementary Figure S3**). Strikingly, IL-5Rα^+^ eosinophils were only temporary reduced with the treatment at T16 (**Figure 3D**), with MFI values being unaltered (**Supplementary Figure S3**. Finally, Siglec-8^+^ eosinophils decreased after the therapy at early time point (T4), but this was not sustained over a longer period (T16, T32) (**Figure 3F**).

### 3.4 Mepolizumab treatment did not affect the proportion of regulatory eosinophils

**Figure 4:**
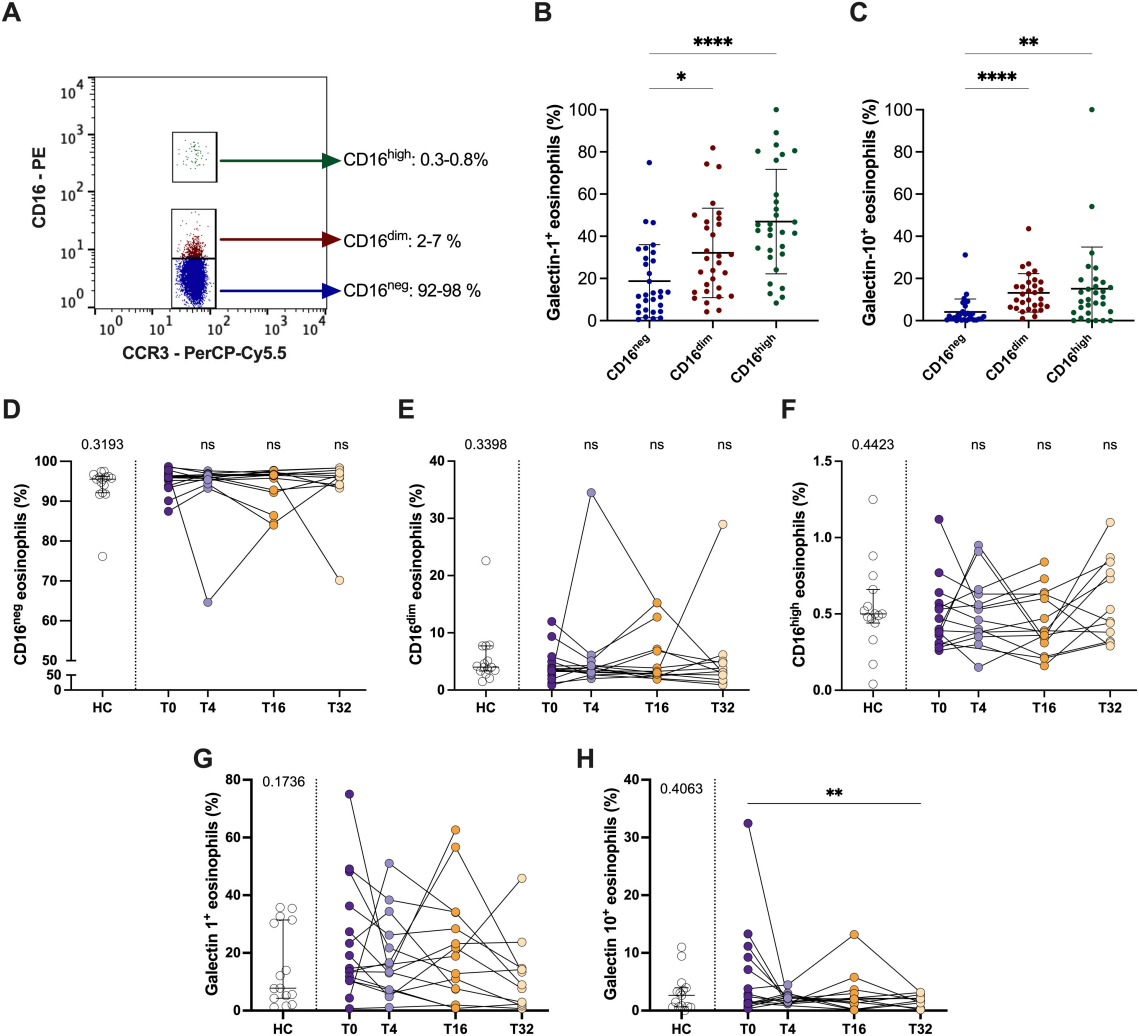
CD16^+^ regulatory eosinophil proportions do not change after mepolizumab treatment. **(A)** CD16^neg^, CD16^dim^ and CD16^hi^ proportions in a representative donor; **(B, C)** galectin-1^+^**(B)** and galectin-10^+^**(C)** eosinophils within the different eosinophil subpopulations, CD16^neg^, CD16^dim^ and CD16^hi^. **(D, E)** Percentage of CD16^neg^, CD16^dim^ and CD16^hi^ eosinophils in healthy (HC) and SEA before (T0) and after 4 (T4), 16 (T16), and 32 (T32) weeks of mepolizumab treatment. **(G, H)** Percentage of galectin-1^+^**(G)** and galectin-10^+^**(H)** eosinophils in healthy (HC) and SEA before (T0) and after 4 (T4), 16 (T16), and 32 (T32) weeks of mepolizumab treatment. Mann-Whitney U test to compare HC vs SEA; p-value is shown for each comparison. A mixed-effects analysis with the Geisser-Greenhouse correction followed by Holm-Šídák’s multiple comparisons test was used to address the effect of mepolizumab treatment at different time-points; *p < 0.05; **p < 0.01; ***p < 0.001.

Next, we evaluated if mepolizumab induced changes in the proportions of regulatory eosinophils or the levels of galectin-1/-10. First, we investigated the levels of the eosinophil subpopulations based on their CD16 expression in peripheral blood. We found two distinct subpopulations of CD16^+^ eosinophils: CD16^dim^ (2-7% of total eosinophils) and CD16^high^ (0.3-0.8% of total eosinophils) (**Figure 4A**). Galectin-1 and galectin-10 expression was higher in both subpopulations of eosinophils compared to CD16^neg^ cells, reinforcing their role as regulatory eosinophils (**Figures 4B, C**). Interestingly, mepolizumab did not affect the proportion of CD16^+^ eosinophils (**Figures 4D–F**) or galectin-1 (**Figure 4G**), although galectin-10^+^ eosinophils drop at week 32 (**Figure 4H**).

### 3.5 Mepolizumab treatment reduced the percentage of inflammatory eosinophils in patients with SEA

**Figure 5:**
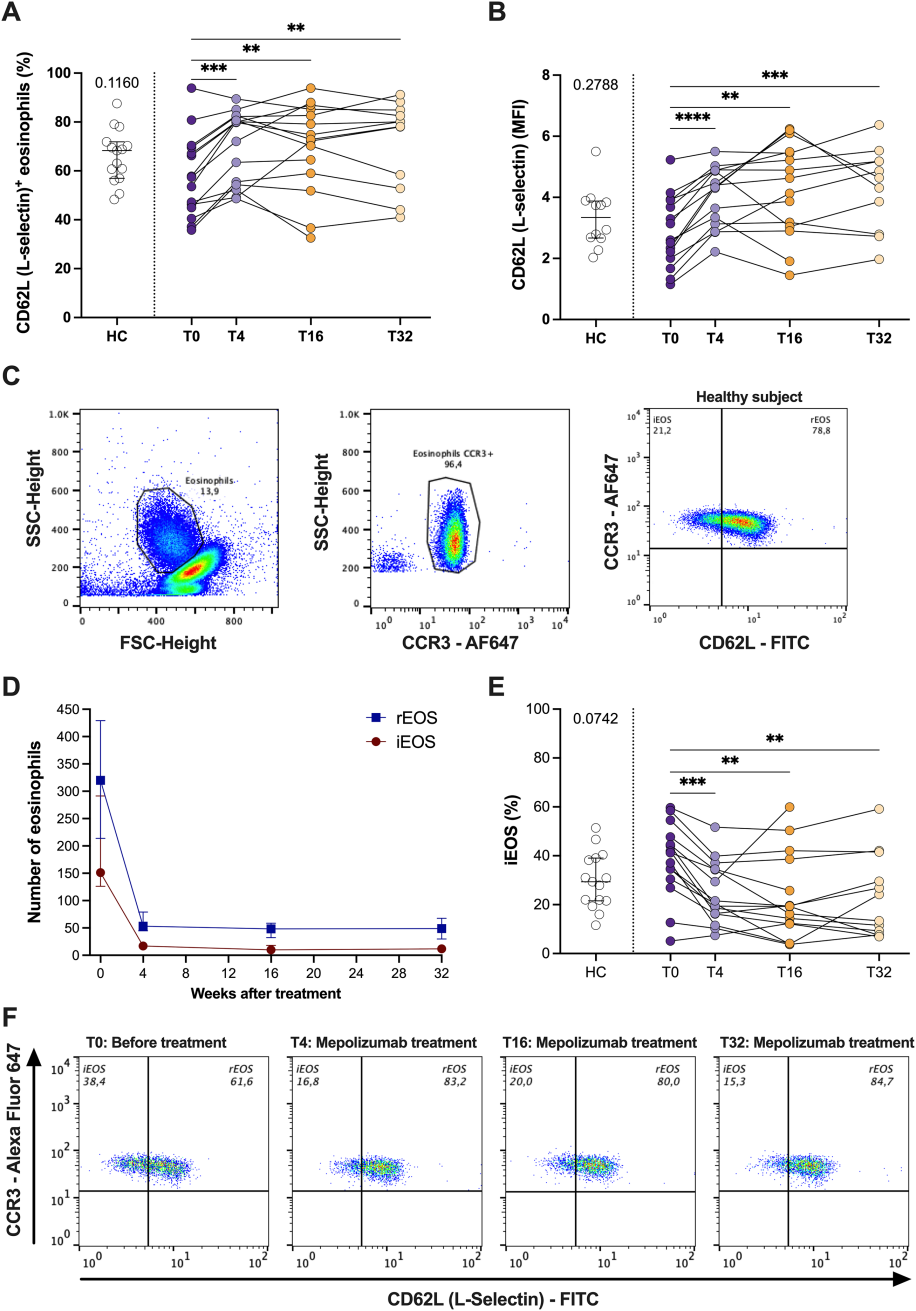
Mepolizumab treatment modifies the functional phenotype of blood eosinophils. **(A, B)** Expression of CD62L (L-selectin) in healthy (HC) and SEA before (T0) and after 4 (T4), 16 (T16), and 32 (T32) weeks of mepolizumab treatment in terms of % of + cells **(A)** or MFI **(B)**; **(C)** iEOS (CD62L^lo^) and rEOS (CD62L^hi^) eosinophils can be distinguish in peripheral blood and they are modified with mepolizumab treatment. **(D)** Change in the number of iEOS and rEOS with mepolizumab treatment. **(E)** Percentage of iEOS in healthy (HC) and SEA before (T0) and after 4 (T4), 16 (T16), and 32 (T32) weeks of mepolizumab treatment. **(F)** Changes in iEOS (CD62Llow) and rEOS (CD62L^hi^) percentages with mepolizumab treatment. A representative donor was selected. Mann-Whitney U test to compare HC vs SEA; p-value is shown for each comparison. A mixed-effects analysis with the Geisser-Greenhouse correction followed by Holm-Šídák’s multiple comparisons test was used to address the effect of mepolizumab treatment at different time-points; *p < 0.05; **p < 0.01; ***p < 0.001.

Then, we studied how mepolizumab treatment affect the expression of CD62L (L-selectin), a molecule previously associated to changes in the function of eosinophils (19). We have found an upregulation of CD62L after treatment (both % and MFI value) (**Figures 5A, B**). Moreover, our analyses revealed the presence of two eosinophils sub-phenotypes based on CD62L expression: iEOS (CD62L^lo^) and rEOS (CD62L^hi^) (**Figure 5C**). Mepolizumab treatment resulted in a decreased number of both eosinophil subpopulations (**Figure 5D**). However, the balance of them changed with the treatment (**Figures 5E,F**). Thus, the percentage of iEOS was lower than before treatment and similar to that of healthy subjects (**Figures 5E, F**). Furthermore, this proportion was sustained over the whole treatment period (T4-T32) (**Figures 5D, F**). The opposite pattern was found for the percentage of rEOS (**Figure 5F**). A correlation analysis was performed between changes in iEOS before and after mepolizumab and clinical outcomes with no significant associations (ΔiEOS vs. ΔACT: r = –0.02, p = 0.53; ΔiEOS vs. ΔFEV1%: r = 0.51, p = 0.067).

### 3.6 sCD62L (sL-selectin) levels in serum were reduced by mepolizumab treatment and associated to the number of rEOS

**Figure 6:**
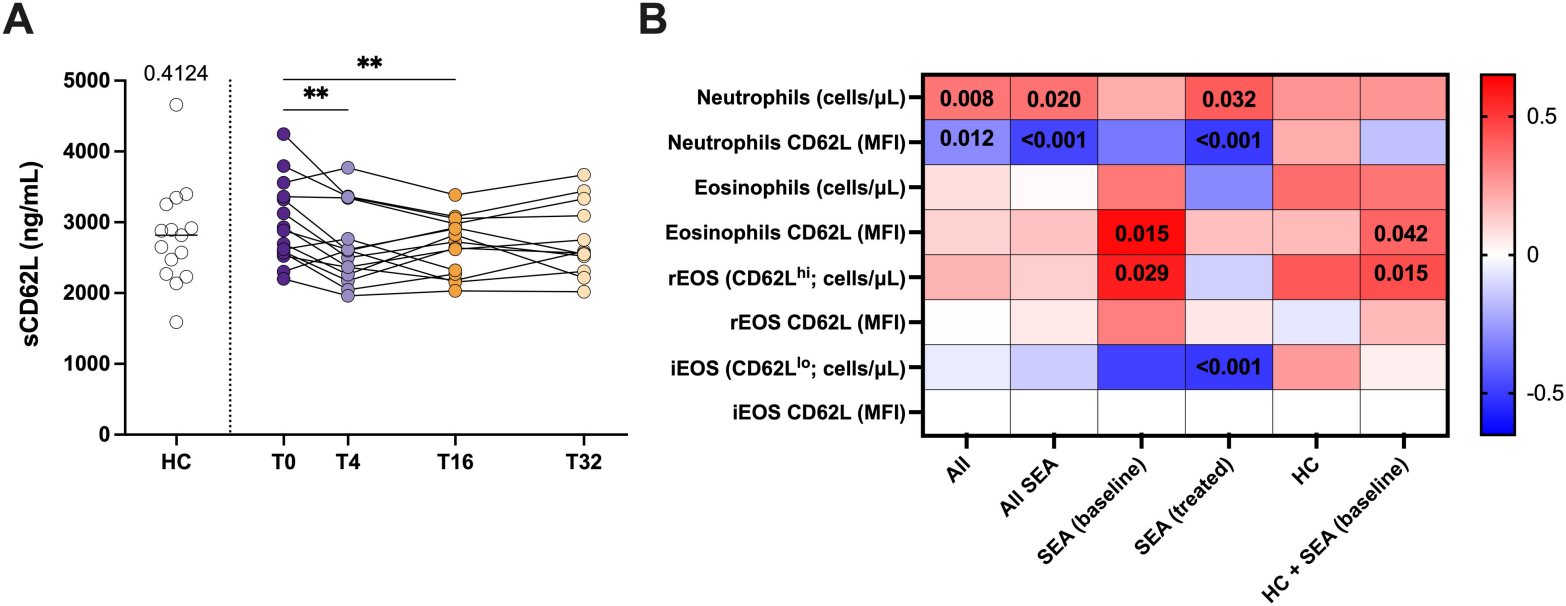
Mepolizumab treatment decreases the levels of sCD62L in serum. **(A)** Levels of sCD62L (sL-selectin) in serum from healthy (HC) and SEA before (T0) and after 4 (T4), 16 (T16), and 32 (T32) weeks of mepolizumab treatment; Mann-Whitney U test to compare HC vs SEA; p-value is shown for each comparison. A mixed-effects analysis with the Geisser-Greenhouse correction followed by Holm-Šídák’s multiple comparisons test was used to address the effect of mepolizumab treatment at different time-points; *p < 0.05; **p < 0.01; ***p < 0.001. **(B)** Heatmap plot showing the correlation of sCD62L levels in serum with the number of neutrophils, eosinophils, iEOS (CD62L^lo^), and rEOS (CD62L^hi^), and their CD62L surface expression (MFI). iEOS are negative for CD62L; thus, no MFI values were included. Spearman test was used for correlation. Significant p-values (< 0.05) are depicted.

We wanted to address if the changes in the number and proportion of iEOS/rEOS in response to mepolizumab treatment are reflected in serum. For that we have measured the levels of sCD62L (sL-selectin). As shown in **Figure 6A**, we could not find significant changes in SEA compared to HC, but sCD62L levels in serum clearly drop after 4 weeks of treatment (**Figure 6A**). sCD62L levels positively correlate with the number of neutrophils (100% positive for CD62L), but inversely with their expression (MFI) (**Figure 6B**). However, neither the number of neutrophils nor their CD62L surface expression were affected by mepolizumab treatment (**Supplementary Figure S4**; **Supplementary Table SII**). Strikingly, sCD62L levels were not correlated with total number of peripheral blood eosinophils, nor with the number of iEOS (CD62L^lo^) (**Figure 6B**). However, a significant and positive correlation was found with the expression of CD62L in the surface of eosinophils and with the number of rEOS in SEA patients (**Figure 6B**). Overall, our results suggest that the decrease in sCD62L levels in serum might reflect the reduction of the number of rEOS after the treatment.

### 3.7 Gene expression profiling revealed that protein changes were not always accompanied by mRNA alterations

**Figure 7:**
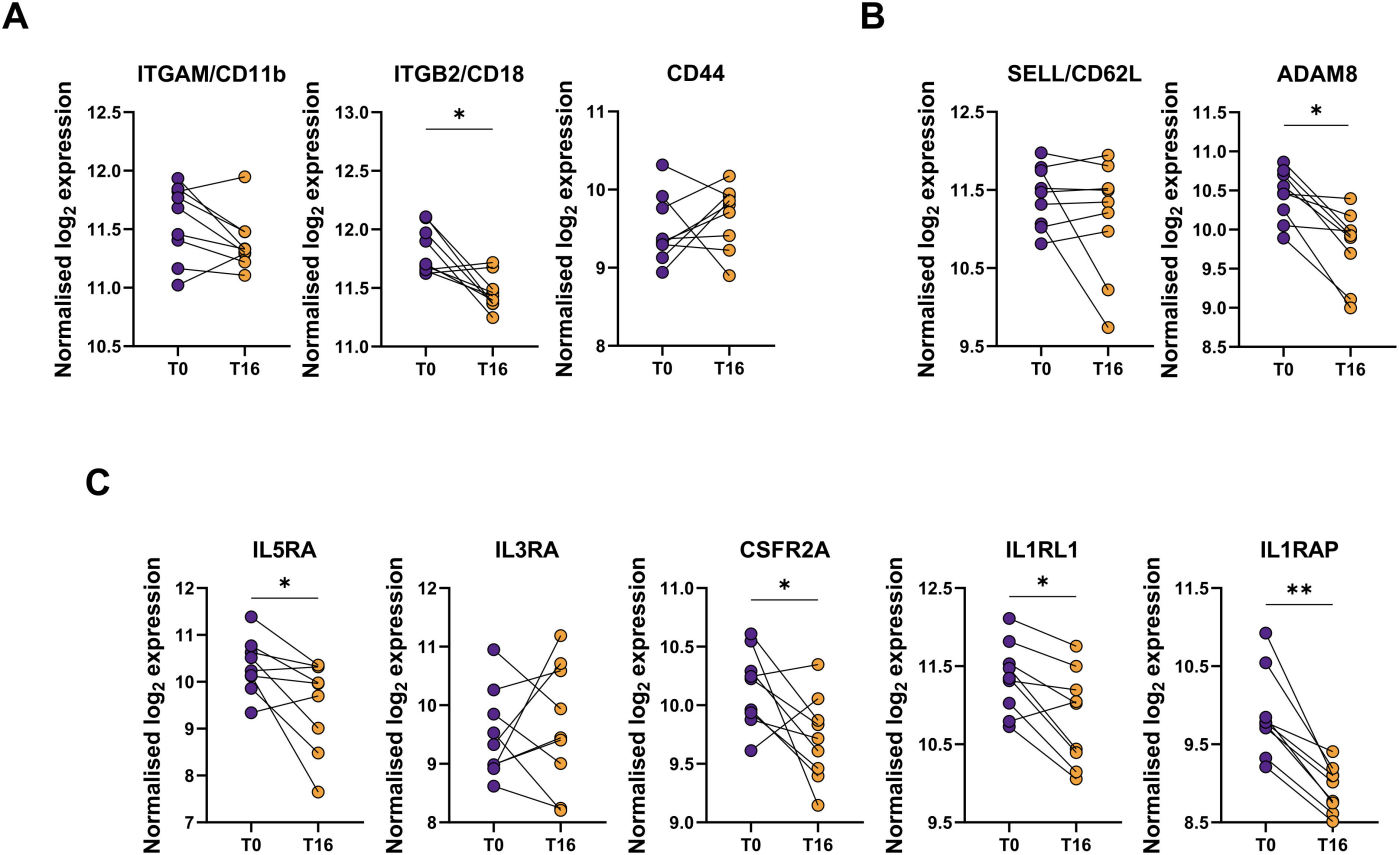
mRNA gene expression does not always follow the same pattern as the protein surface. mRNA expression of selected targets related to **(A)** activation markers (*ITGAM*, *ITGB2*, *CD44*); **(B)** CD62L and its shedding (*SELL, ADAM8*); and **(C)** cytokine receptors (*IL5RA, IL3RA, CSFR2A, IL1RL1, IL1RAP*) were measured by Nanostring analysis. Raw counts were normalized using top 18 stable housekeeping genes. Normalized gene expression was log_2_ transformed. A paired differential expression analysis comparing T0 and T16 was performed. p-values were adjusted for multiple testing using the Benjamini-Hochberg correction. *p < 0.05; **p < 0.01; ***p < 0.001.

Finally, we evaluated if the changes in the surface protein abundances were reflected in mRNA expression. To accomplish this, we compared the gene expression of targets analyzed at surface protein level, before and after 16 weeks of mepolizumab, as well as others functionally related. *ITGAM*, encoding for the CD11b protein, and *CD44* mRNAs were not downregulated; however, *ITGB2* (CD11b coreceptor) showed decreased expression (**Figure 7A**). Unlike the clear upregulation of CD62L surface protein abundance, its corresponding *SELL* transcripts were not downregulated at mRNA level. Remarkably, *ADAM8*, encoding for a metalloproteinase involved in CD62L shedding (21), showed decreased levels following mepolizumab (**Figure 7B**). Finally, we examined the mRNA levels of receptors against activating eosinophil signals. *IL5R* was reduced after 16 weeks validating its decrease at protein level at the same timepoint; similar reductions were reported for the α subunit of GM-CSFR (i.e., *CSFR2A*) and the components of IL-33R (i.e., *IL1RL1* and *IL1RAP*). Conversely, *IL3R* mRNA was not changed despite the lower surface protein levels (**Figure 7C**). Altogether, this points to transcription-independent regulation mechanisms (e.g., internalization, shedding) driving the altered surface protein abundances.

## 4 Discussion

In the present work, we performed a flow cytometry study to investigate the activation and functional phenotype of eosinophils in SEA compared to HC (transversal study) and in response to mepolizumab treatment (longitudinal study). At baseline, eosinophils from SEA patients showed a higher activation profile compared to HC, represented by a higher percentage of CD11b^+^, CD44^+^, and IL-5Rα^+^ eosinophils. Mepolizumab treatment improve asthma control with no changes in pulmonary function. Furthermore, a reduction in the number of basophils and eosinophils in blood was found after anti-IL5 therapy. Interestingly, mepolizumab decreased the activation pattern of the remaining eosinophils, with a clear down-modulation on surface expression of CD44 and IL-3Rα already at week 4. The functional phenotype of the remaining eosinophils was also modified with the treatment, showing a reduction of iEOS (CD62L^lo^) percentage without affecting the balance of regulatory subpopulations. Finally, although the number of both iEOS (CD62L^lo^) and rEOS (CD62L^hi^) descend with the treatment, only rEOS were associated to a decrease in sCD62L.

In this work, we have performed a real-world study of the effect of mepolizumab in SEA. Our patient cohort displayed a mean blood eosinophil count of 541 ± 249 eosinophils/μL; higher than the 250–300 eosinophils/μL observed in the DREAM, MENSA, and MUSCA clinical studies (8–10), and more resemblant to the works from routine clinical practice (12–14, 22–24). In line with these studies, the number of eosinophils rapidly decrease after 4 weeks of treatment (81 ± 34 eosinophils/μL), maintaining similar levels until the last visit. The decrease in eosinophil count was accompanied by an improvement in asthma control (ACT score), but no changes in pulmonary function. Previous real-world mepolizumab studies do report a significant increase in FEV1 (13, 23), but they were based in much larger patient cohorts. Another factor influencing the lack of effect on lung function could be the good lung function to start (mean FEV1 of 87.8% predicted).

A change in the number of eosinophils in response to mepolizumab treatment is of high relevance, but it is not always associated with an improvement in asthma symptoms (15). Therefore, it is also important to study the functional phenotype of the remaining eosinophils after the therapy. First, we have evaluated the activation pattern of eosinophils in SEA before and after blocking IL-5 for a short (4 weeks) and long (16 and 32 weeks) period. We measured the expression of two traditionally activation markers (16), CD11b (αM integrin) and CD44. Despite no changes were previously found in asthma compared to healthy (16), our results demonstrated a significantly higher surface expression of CD11b and CD44 on eosinophils from SEA patients. Previous studies evidenced that IL-5 upregulates the levels of CD11b and CD44 on human eosinophils (16) which, together with the increased serum IL-5 levels found in severe vs. mild asthma (7), could explain the upregulation of these markers in our cohort of SEA patients. Interestingly, mepolizumab treatment significantly decreased the levels of CD44 at T4, and this was maintained through the whole treatment period (T4-T32). Our results contrast with those from Kelly et al., who did not find changes in CD44 or other IL-5 dependent activation markers (i.e., CD69 and CD23) (25). However, these differences could be related to a different experimental set up (single i.v. dose of 750 mg mepolizumab; 1 month of therapy); our results come from a real-life clinical setting using an approved dose of mepolizumab (100 mg, s.c., every 4 weeks during 32 weeks). Although the % of CD11b^+^ eosinophils remained unaltered after mepolizumab treatment, MFI values were decreased; as previously shown by Luo et al. (26).

Similar to CD11b and CD44, we also found an upregulation of IL-5Rα in patients with SEA, which reinforces the use of mepolizumab (anti-IL5 mAb) in this group. After mepolizumab, we found a decrease after 16 weeks of treatment which was also observed at mRNA level, although IL-5Rα^+^ eosinophil percentage was restored at later time-point (T32). Indeed, previous reports in asthma or other eosinophilic diseases have shown no changes in IL-5Rα expression on circulating eosinophils after mepolizumab treatment, or even increased levels (24–27). On the other hand, IL-3Rα was markedly reduced with anti-IL5 treatment, consistent with previous studies (25–29). Yoshimura-Uchiyama et al. demonstrated that IL-5 significantly increases IL-3Rα expression *in vitro*, exhibiting an even stronger effect than IL-3 itself (30). Consequently, by blocking IL-5 binding to IL-5Rα, mepolizumab not only inhibit IL-5-dependent eosinophil activation/function, but also reduces IL-3-dependent features through a bystander effect that lowers IL-3Rα levels. The restriction of IL-3 pathway is of particular importance since this interleukin not only plays overlapping roles (e.g. survival/adhesion) with IL-5, but it is also involved in early differentiation stages of eosinophil biology (31), and exerts a stronger effect in eosinophil degranulation and chemotaxis (32). Therefore, the combined inhibition of IL-5 and IL-3-dependent pathways by mepolizumab underscores its broader therapeutic impact on eosinophil regulation and its potential to mitigate both chronic inflammation and eosinophil-driven tissue damage.

Siglec-8, a marker of mature eosinophils (33), was also affected by mepolizumab treatment. In this regard, we observed a decrease in Siglec-8^+^ eosinophils after 4 weeks of treatment, but this effect was not sustained at 16 or 32 weeks. Arakawa et al. have found an up-regulation of Siglec-8 expression with IL-5 *in vitro*, followed by a down-modulation in response to mepolizumab, aligning with our results (34). Although the transient pattern is intriguing, these changes may reflect dynamic changes in eosinophil biology during treatment. The reestablishment of Siglec-8^+^ eosinophil levels at later time points might indicate shifts in eosinophil phenotypes, with potential implications for eosinophil function and response to therapy. Further investigation is warranted to elucidate the mechanisms and clinical significance of these observations.

CD62L is another protein constitutively expressed in blood eosinophils previously associated to eosinophil activation. Indeed, important mediators of SEA, including IL-5, GM-CSF and TSLP, are able to induce shedding (i.e., the downregulation) of CD62L on human eosinophils (16, 35–37). We found a clear upregulation of CD62L expression after mepolizumab treatment in peripheral blood eosinophils from SEA patients. Hassani et al, found a similar increase comparing mepolizumab treated patients with those receiving placebo (29). As firstly described by Mesnil et al. (19), we could also differentiate two eosinophils sub-populations based on the expression of CD62L: iEOS (CD62L^lo^) and rEOS (CD62L^high^). iEOS were reported to have a higher expression of proinflammatory genes, and an increased production of ROS (19, 38). On the other hand, rEOS were previously associated to tissue homeostasis, with potential functions associated to tissue repair and regeneration (26, 38–41). In the present work, we could find a clear trend (p = 0.0742) towards an increase of iEOS in SEA vs. HC. In line with this, several mediators of eosinophilic asthma, including HDM, OVA, or IL-33, induce the accumulation of iEOS, but not rEOS in lungs, airways and lymph nodes of mice (19, 42). Cabrera-Lopez et al. have also described a higher proportion of iEOS (independent of total number of eosinophils) in asthma compared with healthy or COPD donors (43). A higher proportion of iEOS was also previously reported in blood and nasal polyps of SEA patients (44). Moreover, Vultaggio et al. found a positive correlation of iEOS proportion in SEA patients with the clinical score of asthma, nasal polyps, and the number of exacerbations. They have also found that IL-5 induces the expansion of CD62Llow/iEOS *in vitro* (45). Interestingly, mepolizumab treatment clearly reduced iEOS, and concomitantly enhanced rEOS proportion in our patient’s cohort. This was also confirmed in the recently published studies from Vultaggio et al. (45) and Fricker et al. (46). Furthermore, eosinophils from healthy and mepolizumab treated patients did not differ each other, suggesting that residual eosinophils resemble the phenotype of healthy subjects (47). However, we were not able not find a significant correlation between ACT score and the numbers of eosinophil phenotypes. This is probably due to the limited samples size and other multiple factors which altogether contribute to achieve asthma control.

Soluble L-selectin in serum was also found decreased with mepolizumab treatment (48), but more likely associated to the decrease in the number of rEOS after treatment. Indeed, we found a positive correlation of rEOS (number) with the levels of L-selectin in serum in patients with SEA. Consistent with this, ADAM8, a metalloproteinase related with CD62L shedding (21), showed decreased mRNA levels after 16 weeks of treatment. It is important to notice that although rEOS were previously defined as eosinophils with homeostatic functions, there is some controversy. Wilson et al. (49) have suggested the existence of three different subpopulations of sputum eosinophils in children with SEA: CD62L^lo^, CD62L^int^, and CD62L^hi^; the last two specifically associated to children that experienced disease exacerbations despite mepolizumab treatment. Interestingly, phenotypic characterization of CD62L^+^ subpopulations (likely rEOS) in sputum indicated a higher activation marker and EPX expression (49). However, these results need to be interpreted cautiously and taking into account the different compartments (i.e., sputum vs blood) and study population (i.e., children vs adults).

Finally, Davoine et al. identified a subpopulation of CD16^+^ eosinophils in the peripheral blood of healthy individuals and allergic asthma patients, reporting an increase in this subset within the allergic asthma group (50). More recently, two works from Lingblom et al. suggested the presence of a subpopulation of CD16^+^ eosinophils with T cell suppressive features (18, 51). CD16^+^ eosinophils exert their regulatory capacity through galectin-10 (18, 51). Similarly, we found two clearly distinct subpopulations of CD16^+^ eosinophils: CD16^dim^ and CD16^hi^; both with higher Galectin-10 expression compared to CD16^neg^ cells. Interestingly, CD16^+^ subpopulations of eosinophils were not changed in SEA vs. healthy or affected by mepolizumab treatment. However, we could find a decrease in galectin-10^+^ eosinophils at week 32, which could explain the reduction in serum galectin-10 levels after mepolizumab treatment, previously reported by Kobayashi et al. (52). Galectin-1, another protein with regulatory functions (53), was also enriched in CD16^+^ eosinophil subpopulations, but its levels did not change after treatment.

As any other work, our study has some limitations. The changes in eosinophil subpopulations and their activity after mepolizumab treatment are accompanied by an improvement in ACT, suggesting their potential use as biomarkers of response. Nevertheless, the small number of patients initiating mepolizumab treatment (N = 15) limited the possibility to detect non-responders, and how the EOS activity is on those patients, but our work lays the foundation for larger studies to comprehensively evaluate the role of eosinophil activity and other factors in clinical outcomes. Also, we have no data on impact on exacerbation as the study is too short and numbers too small, but that was not the purpose of this work. Regarding our clinical population study, the lack of a placebo to compare our results implies another limitation, but our data come from routine clinical settings. In addition, the flow cytometer used only allow us to simultaneously detect 4 proteins, which prevent the evaluation of different markers within the studied eosinophil subpopulations.

## 5 Conclusions

Mepolizumab treatment drastically decreases the number, but also the activation status of peripheral blood eosinophils. In addition, this therapy modifies the functional phenotype of eosinophils, with a reduction of iEOS (CD62L^lo^ eosinophils) percentage without affecting the balance of regulatory subpopulations (CD16^+^ eosinophils).

## Data Availability

The raw data supporting the conclusions of this article will be made available by the authors, without undue reservation.
