# Supplementary material for: Mepolizumab treatment alters the functional phenotype of eosinophils in severe eosinophilic asthma
Source: Front Immunol. 2025 Nov 27;16:1654111. doi: 10.3389/fimmu.2025.1654111 (PMC12695602; doi:10.3389/fimmu.2025.1654111)
Supplement: Supplementary file 1 [file DataSheet1.docx]

Supplementary Material

# Supplementary Methods

ERS/ATS criteria were used to diagnose severe uncontrolled asthma. Accordingly, this disease group was defined as asthma patients requiring treatment with high-dose ICS plus LABA for the previous year, or systemic corticosteroids for ≥50% of the previous year, yet remaining uncontrolled. Uncontrolled asthma was defined by at least one of the following:

- Poor symptom control (ACQ consistently ≥1.5, ACT <20, or not “well-controlled” by NAEPP/GINA guidelines).
- Frequent severe exacerbations: ≥2 bursts of systemic corticosteroids (≥3 days each) in the previous year.
- Serious exacerbations: ≥1 hospitalization, ICU stay, or mechanical ventilation in the previous year.
- Airflow limitation: post-bronchodilator FEV1 <80% predicted with reduced FEV1/FVC below the lower limit of normal.

# Supplementary Figures and Tables

## Supplementary Figures

**
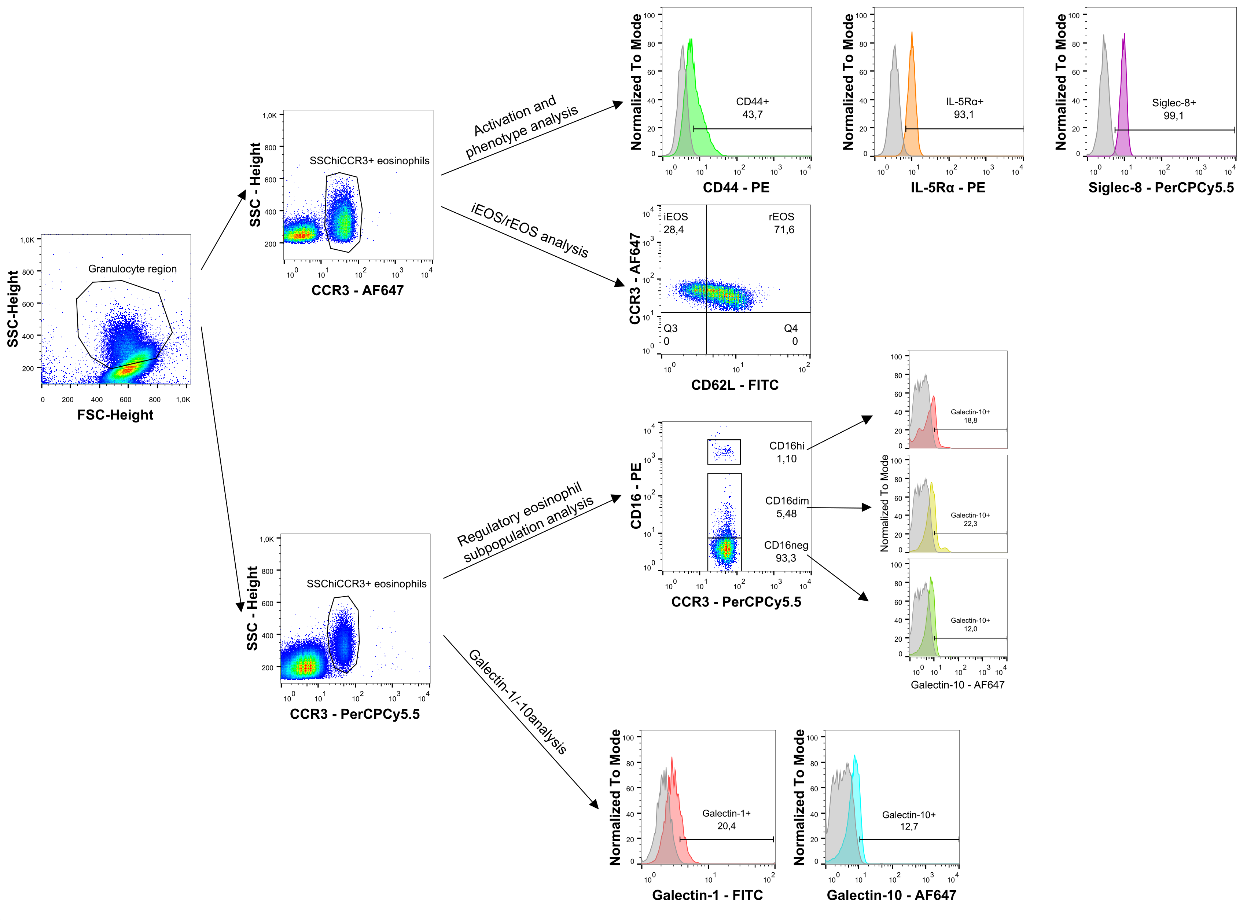
**

**Figure S1: Gating strategy for the analysis of eosinophils and their subpopulations**. Eosinophils were gated as granulocyte-like potential cells based on FSC/SSC and their positive expression of CCR3 (either on AF647 or PerCPCy5.5 channel). Next, we examined the eosinophil activation and phenotype status [CD44, IL-5Rα, Siglec-8 (and CD11b, CD62L and IL-3Rα, not shown in this depiction)], the number and proportion of iEOS/rEOS and regulatory eosinophils and the levels of galectin-1/-10 both in the total eosinophil subpopulation and the regulatory eosinophil subpopulations.


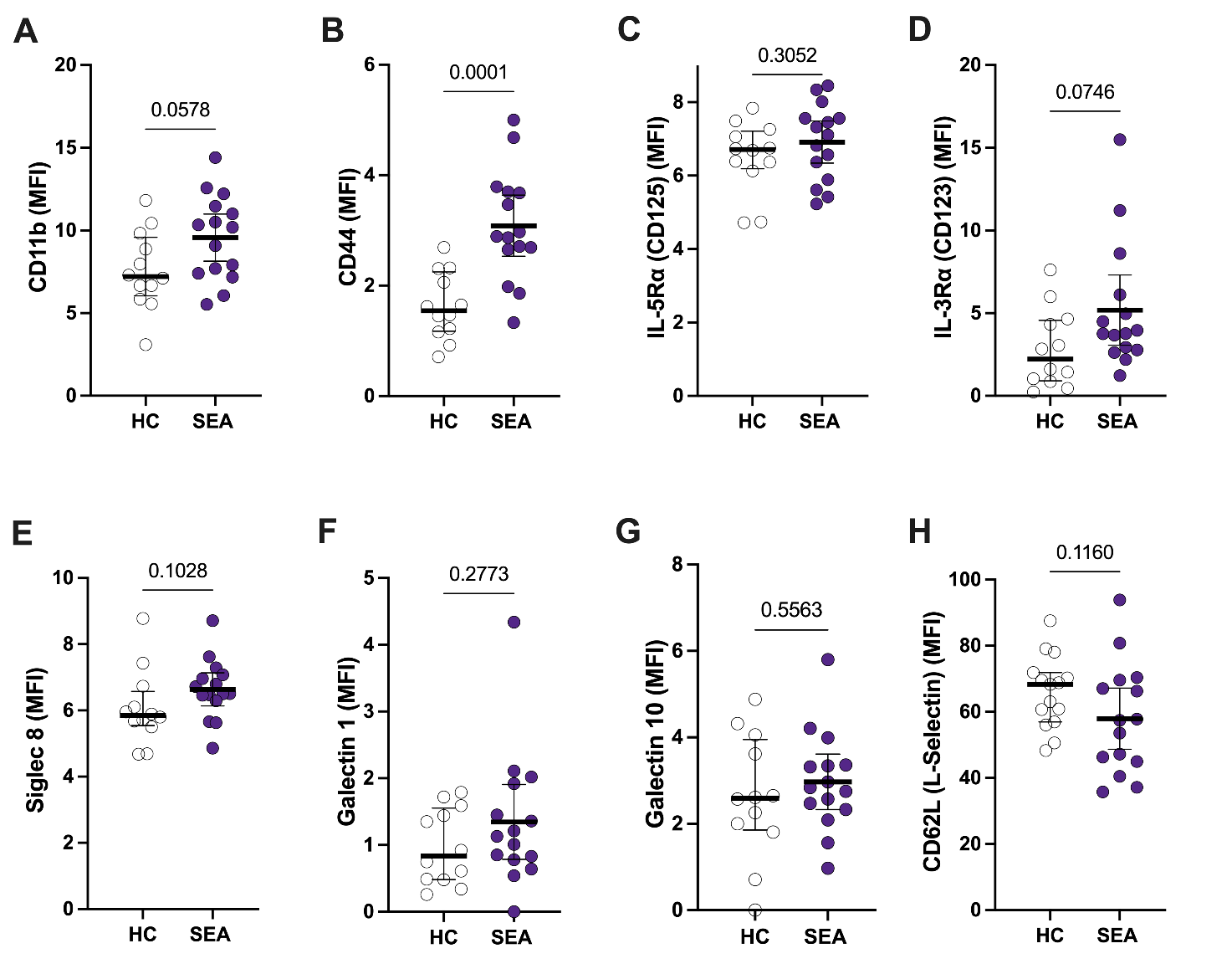


**Figure S2: Peripheral blood eosinophils from SEA patients show a different activation profile compared to HC**. The median fluorescence intensity (MFI) of CD11b (A), CD44 (B), IL-5Rα (C), IL-3Rα (C), Siglec 8 (E), galectin-1 (F), galectin-10 (G), and CD62L (L-selectin) (H) in eosinophils is depicted. Mann-Whitney U test; p-value is shown for each comparison.


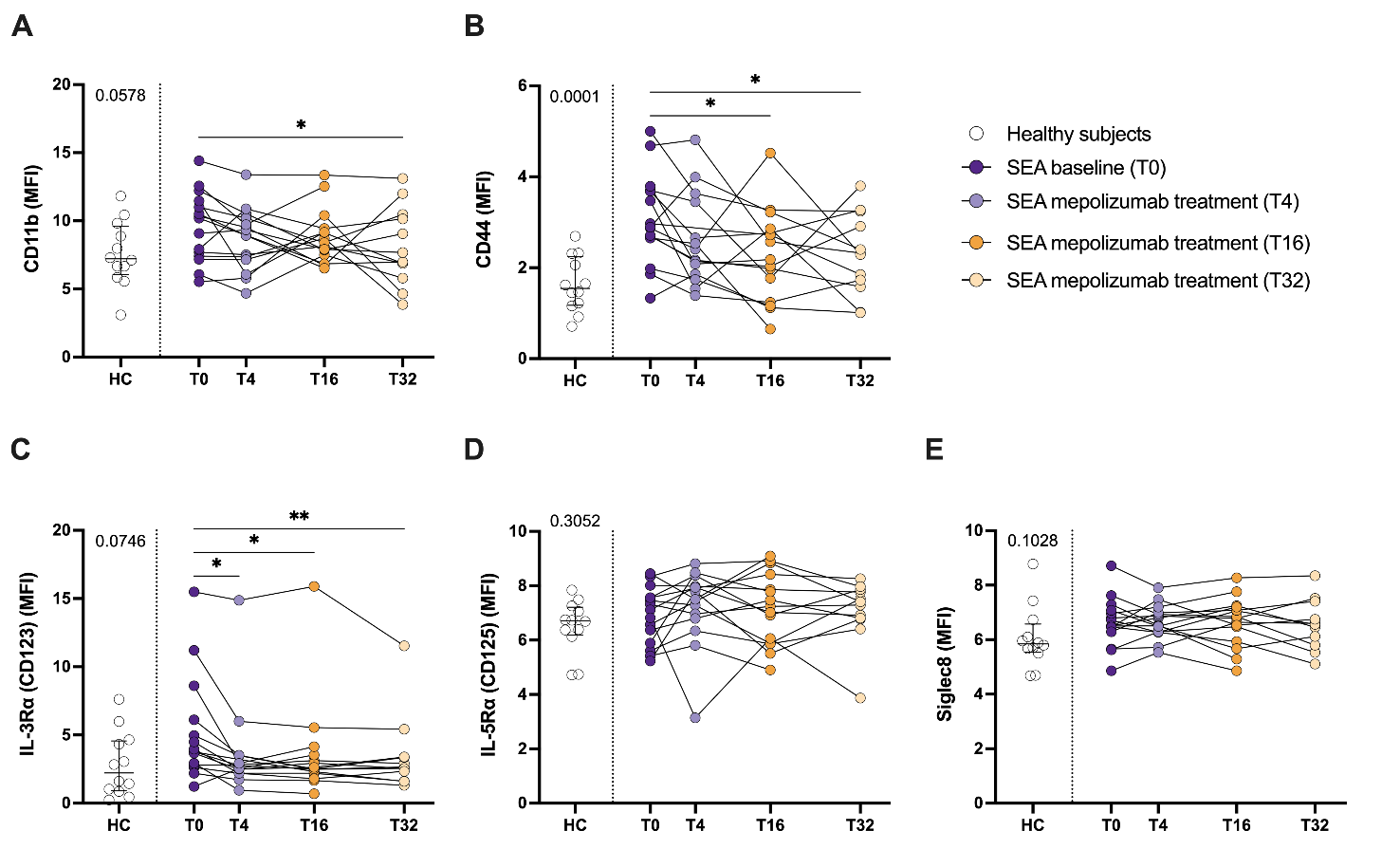


**Figure S3: Peripheral blood eosinophils have a lower activation status after mepolizumab treatment.** The median fluorescence intensity (MFI) of CD11b (A), CD44 (B), IL-3Rα (C), IL-5Rα (D), and Siglec 8 (E) in eosinophils in healthy (HC) and SEA before (T0) and after 4 (T4), 16 (T16), and 32 (T32) weeks of mepolizumab treatment. Mann-Whitney U test to compare HC vs SEA; p-value is shown for each comparison. A mixed-effects analysis with the Geisser-Greenhouse correction followed by Holm-Šídák's multiple comparisons test was used to address the effect of mepolizumab treatment at different time-points; *p < 0.05; **p < 0.01; *** p < 0.001.


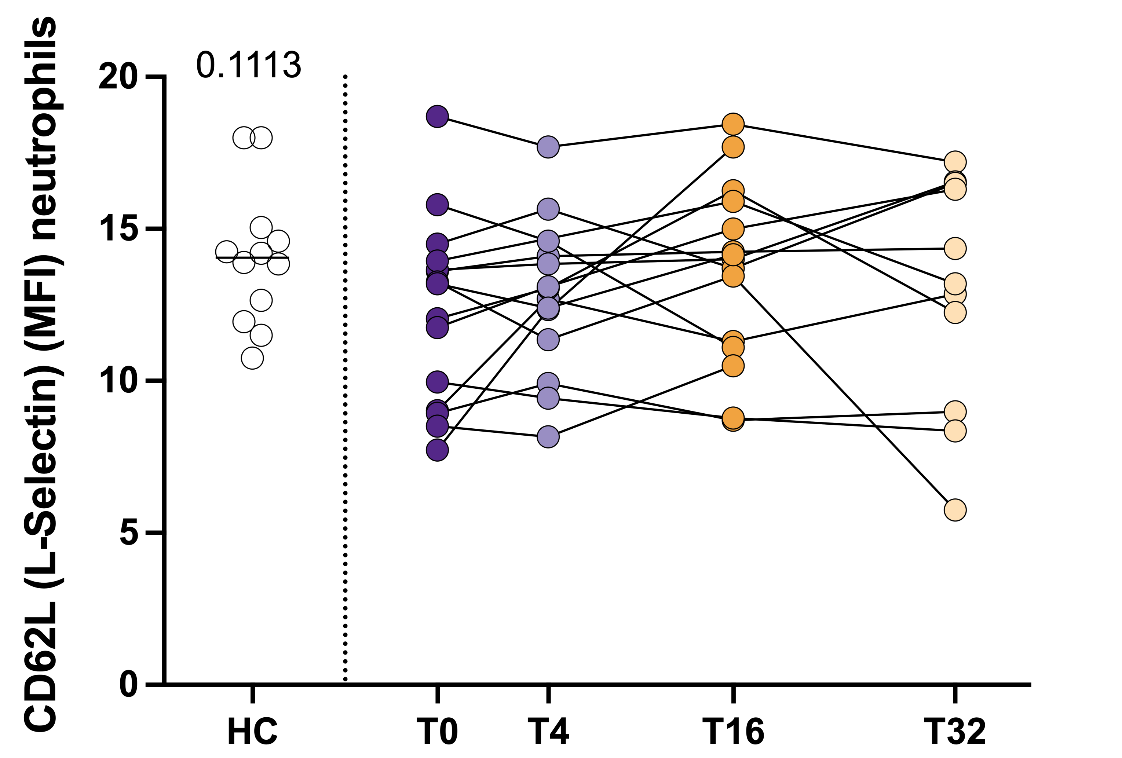


**Figure S4: Mepolizumab did not alter CD62L expression in peripheral blood neutrophils.** (A) The median fluorescence intensity (MFI) of CD62L (A) in neutrophils in healthy (HC) and SEA before (T0) and after 4 (T4), 16 (T16), and 32 (T32) weeks of mepolizumab treatment. Mann-Whitney U test to compare HC vs SEA; p-value is shown for each comparison. A mixed-effects analysis with the Geisser-Greenhouse correction followed by Holm-Šídák's multiple comparisons test was used to address the effect of mepolizumab treatment at different time-points; *p < 0.05; **p < 0.01; *** p < 0.001.

| Table S1. List of antibodies used for whole blood flow cytometry analysis | | | | |
| --- | --- | --- | --- | --- |
| Tube | Target (Fluorochrome) | Panel name | Amount (μL/tube) | Supplier (Catalogue no.) |
| 1 | CD11b (FITC) | Single Positive | 5 | BD BioScience (#562793) |
| 2 | CD16 (PE) | Single Positive | 20 | BD BioScience (#555407) |
| 3 | CCR3 (PerCP Cy5.5) | Single Positive | 5 | BD BioScience (#564189) |
| 4 | CCR3 (Alexa Fluor-647) | Single Positive | 5 | Biolegend (#310710) |
| 5 | Isotype (FITC) | Isotype | 20 | BD BioScience (#555748) |
|  | Isotype (PE) |  | 20 | BD BioScience (#555749) |
|  | Isotype (PerCP Cy5.5) |  | 20 | BD BioScience (#552834) |
|  | Isotype (Alexa Fluor-647) |  | 5 | BD BioScience (#557714) |
| 6 | Galectin-1 (FITC) | Panel 1: Regulatory Proteins | 2 | Biotechne (#NBP2-54457F) |
|  | CD16 (PE) |  | 20 | BD BioScience (#555407) |
|  | CCR3 (PerCP Cy5.5) |  | 5 | BD BioScience (#564189) |
|  | Galectin-10 (Alexa Fluor-647) |  | 5 | Biotechne (#FAB5447R-100UG) |
| 7 | CD11b (FITC) | Panel 2: Activation Proteins | 5 | BD BioScience (#562793) |
|  | CD44 (PE) |  | 5 | BD BioScience (#555479) |
|  | CD48 (PerCP Cy5.5) |  | 20 | Biolegend (#336716) |
|  | CCR3 (Alexa Fluor-647) |  | 5 | Biolegend (#310710) |
| 8 | CD62L/L-selectin (FITC) | Panel 3a: Eosinophil subsets | 20 | BD BioScience (#555543) |
|  | IL-5Rα/CD125 (PE) |  | 10 | BD BioScience (#555902) |
|  | Siglec-8 (PerCP Cy5.5) |  | 5 | Biolegend (#347108) |
|  | CCR3 (Alexa Fluor-647) |  | 5 | Biolegend (#310710) |
| 9 | CD62L/L-selectin (FITC) | Panel 3b: Eosinophil subsets | 20 | BD BioScience (#555543) |
|  | IL-3Rα/CD123 (PE) |  | 5 | Biolegend (#306006) |
|  | Siglec-8 (PerCP Cy5.5) |  | 5 | Biolegend (#347108) |
|  | CCR3 (Alexa Fluor-647) |  | 5 | Biolegend (#310710) |

| Table S2: Patients characteristics throughout mepolizumab treatment | | | | |
| --- | --- | --- | --- | --- |
|  | T0 | T4 | T16 | T32 |
| Pulmonary function |  |  |  |  |
| FEV1, % | 87.8 (24.9) | 72.5 (20.0) | 87.1 (21.0) | 97.1 (25.8) |
| FVC, % | 99.1 (19.4) | 93.1 (16.5) | 97.7 (18.7) | 105.3 (19.9) |
| FEV1/FVC | 69.6 (11.5) | 61.4 (9.3) | 68.9 (11.3) | 71.1 (10.8) |
| FeNO | 60.6 (60.3) | 88.9 (86.4) | 37.5 (41.4) | 76.4 (84.0) |
| Asthma control |  |  |  |  |
| ACT score | 13.4 (4.6) | 16.3 (5.7) | 20.1 (5.4) *** | 23.8 (1.5) **** |
| Eosinophils |  |  |  |  |
| Abs | 540.7 (248.8) | 80.7 (33.8) **** | 59.3 (19.8) **** | 65.8 (34.8) **** |
| % | 7.6 (2.9) | 1.1 (0.4) ** | 1.1 (0.5) **** | 1.1 (0.6) **** |
| Neutrophils |  |  |  |  |
| Abs | 3812.7 (982.8) | 3848.3 (1467.4) | 3817.8 (1309.5) | 3831.7 (1693.9) |
| % | 51.8 (7.7) | 54.1 (8.4) | 54.0 (7.3) | 57.4 (8.7) |
| Lymphocytes |  |  |  |  |
| Abs | 2410.0 (667.9) | 2443.3 (726.5) | 2233.3 (571.1) | 2036.7 (504.4) |
| % | 33.1 (7.1) | 35.6 (7.8) | 34.0 (8.9) | 32.2 (6.6) |
| Monocytes |  |  |  |  |
| Abs | 522.1 (177.9) | 560.0 (171.1) | 537.8 (224.9) | 527.5 (172.0) |
| % | 7.2 (2.2) | 8.0 (1.5) | 7.8 (2.2) | 8.4 (2.5) |
| Basophils |  |  |  |  |
| Abs | 60.7 (21.6) | 51.7 (19.4) | 44.4 (14.2) | 30.8 (15.0)** |
| % | 0.8 (0.3) | 0.8 (0.3) | 0.7 (0.2) | 0.5 (0.3)* |
| Abs: Absolute count of blood leukocytes (cells/μL); T0, T4, T16, T32: Weeks since the start of mepolizumab treatment (T0: 0 weeks; T4: 4 weeks; T16: 16 weeks; T32: 32 weeks).  Mean (SD) of variables is represented.  A mixed-effects analysis (REML), with the Geisser-Greenhouse correction was used to compare statistical differences between the groups. Holm-Šídák's post-hoc analysis was used to compare individual differences. A significant difference between a variable at T4, T16, or T32 vs. T0 is depicted: *p < 0.05; **p<0.01; ***p<0.001; ****p<0.0001 | | | | |
